# The meaning of decision latitude in registered nurses’ night work

**DOI:** 10.1080/17482631.2023.2253572

**Published:** 2023-08-31

**Authors:** Annika Lindahl Norberg, Daniel Falkstedt

**Affiliations:** a Institute of Environmental Medicine, Karolinska Institutet, Stockholm, Sweden; b Center for Occupational and Environmental Medicine, Region Stockholm, Stockholm, Sweden

**Keywords:** Decision latitude, DCS model, job strain model, human service occupation, night work

## Abstract

**Purpose:**

For many employees today, the work situation and work content differ from those of the industrial workers that were originally in mind when the well-known demand-control-support model was developed. The aim of this study was to gain a deeper understanding of the meaning of control, i.e., decision latitude, in post-industrial society, using night-working registered nurses as an example.

**Methods:**

As an example of a modern human service occupation in a value-based organization we choose registered nurses. Twenty-nine registered nurses from 11 departments at three different hospitals participated in semi-structured interviews. The analysis used a thematic approach and was deductive, based on an operationalization of decision latitude.

**Results:**

Findings indicate that the specific meaning of decision latitude is influenced by the specific work organization. Moreover, decision latitude appears to interact in a complex way with demands and support at work.

**Conclusions:**

Decision latitude appears to be influenced by the specific work organization. Thus, when we address self-reported decision latitude, it can have different meanings and potentially different effects in different contexts. Moreover, the interface and interplay between the three constructs decision latitude, demand and support seems to be relevant and complex.

## Introduction

One of the most widely used models to understand the impact of organizational and social factors on employee health is the demand-control-support (DCS) model (Johnson et al., 1989; Karasek, 1979). A substantial body of research indicates, in accordance with the theory, that high demands combined with low control can have a negative effect on health (Theorell, 2020). Further, it has been hypothesized that when the combination of high demands and low control is accompanied by a lack of support from supervisors and colleagues, the situation would be even more detrimental to health (Johnson et al., 1989). Moreover, lack of control alone has been shown to predict several detrimental outcomes, e.g., disability retirement (Knardahl et al., 2017) as well as suicide attempt and death (Almroth et al., 2022). Additionally, access to control at work appears to buffer stress (e.g. Lippert & Venechuk, 2020).,

Control, or *decision latitude*, refers to job autonomy and the possibility to exert control in one’s work. Decision latitude includes the two sub-domains skill discretion and decision authority. The former, skill discretion, concerns the employee’s opportunity to develop and use skills and strategies relevant to the work. Decision authority refers to everyday democracy in the workplace (Theorell, 2020), in the sense of the employee’s influence on decisions about what to do and how to do things at work.

In recent years, a discussion has emerged suggesting that the DCS model may not fit all occupations in post-industrial societies as good as it fit the original population of industrial workers doing assembly-line work (Netterstrøm, 2012; Väänänen & Toivanen, 2018). One hypothesis states that job autonomy may also entail demands arising from responsibilities, and therefore, under certain circumstances, decision latitude may be *negatively* associated with psychological well-being. A curvilinear association between job autonomy and well-being has received some support, although ambiguous (Stiglbauer & Kovacs, 2018). Other researchers argue against this. For example, Clausen et al. (2022) found no support for this hypothesis despite a slightly different pattern regarding the relationship between job autonomy and well-being in client-related work compared with other occupations. In summary, the questioned predictive power of the DCS model still appears to hold, while work content as well as the organization of work changes over the decades.

Yet, to draw comprehensive conclusions and enhance the usability of complex findings researchers and practitioners within work environment and safety would benefit from more in-depth knowledge about the nature of decision latitude in various occupational settings today. The present paper intends to contribute to filling this knowledge gap using the example of the registered nurse, which is a profession that has long existed, but has evolved, e.g., with the development of management and work organization.

Corporate governance and work organization has changed from management through formalization and rules towards self-management and organizational models such as value-based healthcare. To a higher degree than before, the healthcare staff themselves are the ones who plan the details of care, designing and delivering comprehensive solutions to meet the patient’s needs (Teisberg et al., 2020). The role of the registered nurse (RN) has thus changed to include increased influence over the care they provide. At the same time, the expectation of the RN is still to be both a kind of spider in the web, overlooking the activities at the ward, and the emotionally present and self-sacrificing comforter (Gordon & Nelson, 2005; Lindahl Norberg & Strand, 2022). At worst, this will lead to a role that is unclear and boundless, with unreasonable expectations. However, the way RNs’ work is organized during night shifts appears to be more satisfactory than day shifts, provided that the ward is properly staffed. According to qualitative studies, nurses appreciate that the night shift includes a higher degree of autonomy, less fragmentation of the work, and often working closely with a few competent colleagues, which in turn lead to opportunities for development of clinical skills (Lindahl Norberg & Strand, 2022; West et al., 2016). Accordingly, we believe that the RN’s work situation is a suitable example for deepening the understanding of what decision latitude may mean in a human service occupation today.

### Aim

The overarching aim of the present study was to understand decision latitude among night working RNs. Specifically, RNs’ narratives of their work content were analysed through the lens of the control domain of the demand-control-support model.

## Methods

The study was carried out as part of a research project entitled “Sustainable working life for night working healthcare staff”, carried out in Sweden. The overall purpose of the project was to deepen the understanding of what night work means for registered nurses, specifically investigating factors that influence RNs’ motivation to remain in a position that includes night shift work. Data were collected through in-depth interviews to be analysed qualitatively. In the present study, we used the same data for analyses with focus on the theoretical concept *decision latitude*. To ensure complete reporting, the Standards for Reporting Qualitative Research (SRQR) guidelines were used (O’Brien et al., 2014).

### Participants

The project included RNs in Stockholm, Sweden. The inclusion criterion was a minimum of 6 months’ experience of a working time schedule including night work. The final study group comprised a total of 29 participants from 11 departments at 3 different hospitals. Their experience of being an RN ranged from one to 38 years (mean 15), their ages ranged from 25 to 65 years (mean 44). Five of the participants were men. All 29 were registered nurses, and 17 of them had a further education with specialization in a particular health care area.

### Data collection

Participants were invited by an email sent from the principal investigator to all registered nurses at a number of departments selected to represent different specialities. Those who accepted to participate responded with an email or a telephone call.

Semi-structured individual interviews were conducted by two researchers with several years’ experience conducting interviews. The interviews took place at an undisturbed location at the participant’s workplace or at the researcher’s office, lasted for 45–60 min, and were recorded on audio file. Subsequently, the interviews were transcribed verbatim by a professional transcribing agency.

### Ethical considerations

The study protocol was approved by the regional research ethics committee in Stockholm, Sweden (approval number 2018/1360–31/5). In addition, the study was approved by the central HR department in the participants’ organization, each unit manager at the relevant units, as well as each participant. Participants provided written informed consent, confirming knowledge of voluntariness, i.e., if they wished, they could withdraw at any time during the interview. Reflecting on one’s work environment can open one’s eyes to negative aspects of the workplace. Therefore, the interviewers—licenced psychologist and licenced psychotherapist, respectively—assessed during the interviews whether the participants needed occupational health care or other support. No such need was detected. The researchers who conducted interviews and analyses have several years of experience in handling qualitative data. All data were kept confidential, and transcriptions were rendered anonymous. Careful consideration was given to the presentation of the results so that no individuals could be identified.

### Data analysis

The data were originally collected for the aim of the overarching project, i.e., to deepen the understanding of what night work means for registered nurses. For the aim of the present study, data were analysed according to Braun and Clarke’s method for thematic analysis (Braun & Clarke, 2006, 2019). During the analysis, data were approached from a critical realist perspective, applying a semantic, low-level interpretation.

Data analysis was deductive, based on decision latitude as operationalized in the six items of the Swedish Demand-Control-Support Questionnaire (Mauss et al., 2018). Consequently, the analysis was guided by six aspects of decision latitude: *Opportunity to learn new things at work, requirements of skills and competence at work, requirements of creativity and initiative at work, whether work is repetitive or varying*, and influence regarding *what to do at work* and *how to do the work*. The first four refer to *skill discretion* and the latter two to *decision authority*, the two sub-dimensions of decision latitude.

The first author (ALN) performed the analysis and discussed the relevance of the results structure with the second author (DF). As a preparation before the analysis, ALN explored the operationalization of the six aspects of decision latitude. The essence of each aspect was summarized, and significant concepts were used as codes in the analysis. In the first step of the analysis, all meaning units in which RNs related to any aspect of this operationalization were selected and coded. Next, the coded meaning units were organized deductively in the six subthemes. Then all coded meaning units were read through to make sure they were placed in the most suitable subtheme. When doubts arose about the fit of a meaning unit, the operationalization was revisited, and the unit was recoded when appropriate. During the next step of the analysis, ALN recoded the meaning units in each domain inductively on a semantic level. In the final step, the semantic codes were developed into a narrative to describe the content of each subtheme at a higher level of interpretation.

### Reflexivity statement

The disciplinary traditions of ALN, responsible for the analyses, need to be accounted for. During her training as a psychologist, specialist in work and organizational psychology, and PhD in psychosocial medicine she became well acquainted with the traditional interpretation of DCS and other established models for understanding occupational ill-health. She is also familiar with problematizing the somewhat overused and eroded concept of stress. The second author, DF, is currently responsible for epidemiological projects investigating the DCS model at the occupational level, and its relation to the occurrence of mental health problems in Sweden’s population of workers.

## Findings

There turned out to be a considerable overlap between the results for the two aspects “skills/competence” and “creativity/initiative” at work. Therefore, these aspects were merged into one theme. The same was true of the aspects regarding influence on what to do at work and how to do the work, so these were also merged into one theme. The results structure thus includes four themes: Learning new things, Skills/competence and creativity/initiative, Repetitiveness, and What to do and how to do it.

The specific organizational context of the night work, differing from the day shift, forms a common backdrop for the RNs decision latitude. Certain aspects of this context were influential on all four themes: competent dialogue with doctors and knowledgeable colleagues, focus on care and nursing including a overview of each patient’s care, and usually sole responsibility for monitoring the patients.

### Learning new things

- Less theoretical learning, but the more clinical insights
- A fragile balance between educational challenge and distressing overload
- The dialogue with the on-call doctor at times became a clinical tutorial

Learning was to some extent of a different nature during the day and the night shifts: Daytime, ward rounds and arranged lectures gave new theoretical knowledge, while the night shift offered practically applied clinical learning. Yet, some expressed that night shifts were poor from a learning perspective. A focus on theoretical learning seems to engender an unquestioned assumption that night work lacks opportunities for learning. One interviewee started out with this notion but then reasoned that night work indeed may entail development opportunities: *“As a recent graduate, I don’t want to work only nights because I still want to learn things and then I learn better during the day because there I get more knowledge, routines and all that./ … /On the other hand, at night I like it because then I* *have to do certain things, I can’t always ask for help. Sure, I get help if I need, but … it’s like: if I have to do this, yes but, then I have to do it. During the day it’s more like ‘can you come and help me …’. So I develop faster at night, and I think that’s great.” (ID 16).*

Night work enhanced RNs’ professional development by requiring them to make independent assessments of patients’ status and needs and make their own decisions. The organization of night work both enabled and forced them to develop this autonomy. Interviewees’ descriptions illustrated a fine line between the consequences of opportunity and pressure, respectively: On one hand progress and satisfaction, and on the other hand a discouraging feeling of an overwhelming responsibility. Yet, some of the stories indicated that being forced to handle new situations may lead to learning even if the conditions did not reflect an optimal work environment. In the interviews, RNs used the word *“challenge”* to describe difficult situations that they had to solve on their own. *“The thing that is still positive I think, after working this long, is that I learn new things all the time. It’s new challenges … well, it’s challenges, and it is positive when you see it as challenges” (ID 10)*. In order to be educational, a situation had to involve some difficulty but, at the same time, the RN was often alone with a great responsibility. Descriptions would thus contain both appreciation and reluctance.

In contrast, stories depicting professional development were more unambiguously positive when the work implied communicating with the physician on duty in combination with the autonomy of night work and continuity of the patient work. *“You often discuss with the on-call … exchange ideas and thoughts … you get to participate in the process a lot, and I like that.” (ID 15)*. From time to time, descriptions give a picture of informal clinical tutorial. In addition, the dialogue with the on-call doctor became particularly informative because the RN could follow the patient’s treatment and condition, which was rarely the case during the day shift.

### Skills/competence and creativity/initiative

- Autonomy both allowed and forced RNs to use skills and initiative
- Focus on care and nursing meant focus on the skills central to the RN’s profession
- Many issues were solved when skills and creativity could be strengthened through discussion with competent colleagues
- To consult the on-call doctor, the RN had to assess the patient’s condition, estimate the degree of urgency, and often suggest measures, i.e., it required both skills and initiative

Competence and problem-solving skills were vital to manage the everyday work as well as more serious events during the night work. Being alone with a lot of responsibility could certainly be felt as burdensome at times when RNs could not trust that there would be someone to help out if needed. *“It’s vulnerable, that’s the absolute worst thing about night work. As long as it goes well, it goes well and then it’s great, but when it sucks, it sucks, like, properly. And times like that it’s tough to work at night because there is no help. You’re very alone.” (ID 16).*

However, several stories painted a positive picture of the autonomy. Interviewees appreciated to be able to solve upcoming issues by themselves. *“What I think is fun with working nights is the patients who need that little extra/ … /To use my clinical eye, to anticipate before something happens, to know the situation and inform the doctors well in advance before anything happens.” (ID 7)*. Unlike the fragmented day shift, they could get an overall picture of each patient. It was also appreciated to be able to focus on nursing and care. In other words, the skills central to the registered nurse’s profession.

RN colleagues were normally accessible at a ward nearby. Since night work requires certain competence, support from colleagues implied discussions which maintain a certain level of knowledge. Interviewees described a creative dialogue with colleagues on determining which assessments and measures should be taken. It may seem like a contradiction that night work is characterized by both solitary work and close collaboration with colleagues, but both the solitary work and the culture of cooperation are based on the same ground of independence and high competence. Discussions with colleagues were also more accessible than with the on-call doctor. *“RN: It should be an emergency situation, then they [the on-call doctor] must come, it’s as simple as that. But you’re a bit reluctant to call … Interviewer: You don’t call just because you have a bit too much to do. RN: No. No absolutely not. Then you simply make sure to solve it yourself.” (ID 29).*

Thus, if no serious incidents arose, the RN did not need to consult a physician to do their job. In serious and difficult cases, however, consultations were made, often over the telephone. A physician was available on-call on the night shift, usually either busy at another department at the hospital, or sleeping. The responsibility lay with the RN to assess whether the case was urgent enough for them to call for the doctor, and this assessment required knowledge and experience. Besides assessing the patient’s condition and describing it in a relevant way, RNs also needed to estimate the degree of urgency. One interviewee, whose on-call doctor was a surgeon, explained: “*If they’re operating, a nurse takes the phone and then it is important that I don’t call them out unnecessarily. I have to prioritize and that’s where the experience and competence come in, like, deciding if he has to come now-now, or can he come in half an hour.” (ID 16).*

Along with a qualified assessment of the patient’s status required of the RN, consultations also invited suggestions for action, since the on-call doctor was often from a different speciality. Such discussions carried a particular sense of equality because the RN’s knowledge and experience were highly valued.

### Repetitiveness

- Responsibility to monitor the patients and the necessity to handle unanticipated events on your own entailed variation
- Variation in tasks also meant handling unfamiliar situations alone
- The appreciated continuity and focus on care and nursing actually meant less variation, in the sense of less fragmented work
- At departments where the patients slept calmly all night the work could be boring

Each patient’s illness trajectory is somewhat different from the other. A variety of working tasks accompanied the responsibility to monitor the patients and assess whether arising medical events could be handled without consulting the doctor. Generally, this variation was appreciated. When it was not, the problem was not the variation per se, but that tasks were too complicated, and no support was available.

Another facet of work variation included the possibility to be involved in the entire care of a patient, and not just a certain part of the department’s work, such as enrolling new patients. *“I appreciate being able to follow the patient from the moment they enter through the door until they leave the emergency room. During the day it’s like … you enter new patients and enter and enter but never get to know what happened to the them or what samples were taken.” (ID 20).*

On the other hand, a larger continuity in the work with the patients and by far less fragmentation of work could be described in the terms of *less* variation. Yet, narratives illustrated that this lack of variation/fragmentation gave the RN a chance to practice the core of the nursing profession. Although the workload could be considerable during the night, this continuity seemed to bring about a feeling of having the time to do a good job. One interviewee related: *“I appreciate this continuity at night very much. Say that you have a patient who has had a miscarriage and is very sad, at night you often have time to sit with her, you have time to talk and comfort and inform and … simply do a better job. While during the day, then there are ten other things waiting so you kind of go in there and feel like ‘please, stop crying … I don’t really have time’. And then you feel unsympathetic. That’s not why I wanted to be a nurse.” (ID 20).*

Finally, night work meant different things at different departments. The interviewed RNs worked with different specialities, and even if the continuity and focus on nursing and care described above seemed to be the reality for some, it did not apply to all. From certain departments, RNs described how patients often slept quite peacefully during most of the night. The work involved repetitive tasks like mixing and distributing antibiotics for several hours. During such a calm period the work could be rather tedious. *“During the day it is much more fun actually, I think, because then the patients are awake and you can talk, joke, you help them with nursing care and then it is much less medication because then you are responsible for fewer patients.” (ID 28).*

### What to do and how to do it

- Day shift often implied a spider-in-the-web service function for RNs, minimizing the own decision authority
- On the contrary, during the night shift RNs’ autonomy and the focus on care and nursing entailed possibility to decide what to do and how to do things
- Sufficient time for communicative care and other core duties of the RN increased an experience of decision authority
- Inadequate access to competent on-call physicians could force the RN to decide what to do and how to do things even beyond their responsibility and competence

Decision authority, i.e., influence on what to do and how to do things, was a prominent feature of night work, according to the interviews. Almost all the stories conveyed a picture of the night shift as the free zone for the RN to autonomously plan their work and prioritize their tasks without being interrupted. Decision authority in night work was particularly illustrated by the narratives of experienced RNs, who had developed their own approach to how things should be done, although also mentioned by younger RNs.

Thus, the RN’s night work included considerable freedom to decide *how* to do things. Regarding the *what*-to-do aspect, this in healthcare is of course strictly regulated by the patient’s condition and the measures stipulated by evidence-based care. Still, in the details of nursing care RNs had substantial freedom concerning *what* to do.

Although night work was the focus of the interviews, several of the RNs included descriptions of daytime work in their stories to illustrate the contrast. Typically, the daytime work was fragmented and tightly structured by the many duties of healthcare: *“Phone ringing, meetings you have to go to, education, meetings … You have to go to care conferences, you have to call and discuss with a social worker, lots of contacts to be made, rounds… I don’t mean that it’s not an important part of the job, but … [At night] you can just, hands on, plan your work yourself and work with the patients.” (ID 10),*

In addition to the fragmentation, the RN’s role during the day shift tended to be like a hub connecting the parts, making the machinery work. This role had little space for decision authority. *“[W]e have to be the link between everyone”* as one interviewee put it, continuing *“I feel that nurses have to do everything on all levels” (ID 13)*. Thus, besides the lesser fragmentation of night work, decision authority was also enhanced by not being disturbed by others’ opinions of what should be done. *“No one is sitting there telling you what to do and how to do it. You do it, and you do it for the patient’s good.” (ID 7).*

Moreover, decision authority included the possibility to decide both in what order things should be done and how much time should be spent on each task. Work was not necessarily less stressful at night, but RNs still reported a sense of sufficient time, partly because the time could be devoted to care and nursing. Interviewees particularly appreciated the opportunity to plan enough time for communicative care, counselling and informing the patients. Time planning with the intention to truly give the patient what they needed also meant smoother work. During the early night, for example, this could be spending a little extra time with a patient exhibiting unease, to prevent anxiety of a large magnitude later.

In contrast to the positive experience of freedom, RNs occasionally had no choice but to decide what to do and how in situations that was beyond their area of formal responsibility and competence. Explicitly, in some departments where on-call doctors often were junior physicians, a serious event may exceed the junior doctor’s competence. In such cases, RNs sometimes needed to step forward and decide what should be done and how. Feeling forced to step beyond the RN’s responsibility and mandate for the sake of the patient was described as quite stressful.

## Discussion

The overarching aim of the present study was to explore decision latitude among night working registered nurses, in order to gain a deeper insight in what decision latitude may look like in a human service occupation today. Our choice of night working RNs was based on an assumption that their work situation and work content differ from that of industrial workers, which were originally in mind when the DCS model was developed. Registered nursing is a modern human service occupation, and RNs are typically working in the context of value-based healthcare. Particularly during the night shift, they are expected to have a complete overview over the activities at the ward, and they are charged with large responsibility not always accompanied with formal authority (Lindahl Norberg & Strand, 2022; West et al., 2016). Being assigned undue responsibility is a complicated work environment issue that concerns, among other things, role stress and threats to the professional identity. In addition, from time to time nurses face a balancing act when communicating with doctors, on the surface conforming with the traditional hierarchy, but essentially violating it by guiding doctors in their work (Caronia et al., 2020).

A four themes’ structure was considered to do the greatest justice to the results, describing the aspects of decision latitude relating to learning, skills and creativity, repetitiveness, and decision authority, respectively. Certain common elements reappeared in the themes, e.g., social support, autonomy, workload, competence, and formal responsibility, which all influence decision latitude.

Whether decision latitude is available for a worker does not only depend on the degree of freedom allowed in the daily work. One of the influencing factors is the workload, which when optimal provides enough time to practice decision latitude. The interviewees in the present study compared night and day shifts, depicting a more flexible situation at night. Although the work could be stressful during the night shift, they felt that there was latitude to, indeed, use their competence and initiative for the benefit of the patient, which in turn provided invaluable job satisfaction. Appreciation that the night shift allows the RN to perform the essential activities of the profession has also been described by West et al. (2016). In a recent study, researchers (Andersen et al., 2022) explicitly asked employees in knowledge and relational work about their connotations of *influence at work*, and found that influence regarding their core work tasks was perceived as particularly important.

Moreover, decision latitude was influenced by whether colleagues were available for support and practical help. Staffing is an organizational factor which relates to both support and demands. Sufficient staffing is a prerequisite for access to support, while understaffing increases the workload (e.g., Garrett, 2008). Understaffing in healthcare seems, however, to be mostly studied as a risk to patient safety.

During the early development of the DCS model, theoretical complexities in the interplay between social support and decision latitude were recognized. Theorell (2020) refers to reflections about peer support being beneficial for the level of decision latitude. Social support has since then been thoroughly studied in the work context, and is typically considered exclusively beneficial. Accordingly, lack of adequate support is a risk factor for work-related ill health, while access to support is a facilitator of well-being.

Yet, the actual meaning behind the term social support may vary (see e.g., Jolly et al., 2021 for an overview), and there are a few efforts to problematize social interaction at work. According to Gray et al. (2020), there are also several negative qualities of social interaction, of which one is imposing social support, e.g., helping another when the resource is not asked for. In our study, decision authority implied that interviewees were able to plan their work without anyone interfering. The night shift contained more autonomy in that sense compared with the day shift, which was similarly reported by West et al. (2016).

Thus, with the capacity of qualitative research to make paradoxes visible, our findings emphasize the diffuse boundary between autonomy and aloneness. Decision latitude implies being left alone enough to have the opportunity to come up with own solutions to a problem or plan what should be done and how. At the same time, lack of support when needed was experienced as a major problem.

Another aspect of the close relationship between social support and decision latitude applies to learning. Our findings suggest that, to be educative, decision latitude beyond one’s competence requires access to adequate support. In other words, the learning aspect of decision latitude is dependent on competence and social support, which in turn are interrelated. In work with a high level of decision latitude, such as the night working RN, the worker is allowed to solve upcoming issues themselves. In our sample, issues that were somewhat challenging were perceived as educative. However, when the issues were highly challenging and the worker was alone, the situation was described as stressful. In other words, working tasks beyond a person’s area or level of competence can be educational, but only if they are not too challenging and if there is access to support. This can be seen as a workplace learning application of the *zone of proximal learning*, initially articulated in a theory of learning in the developing child (Vygotsky, 1978), and more recently also used to understand workplace learning (Billett, 2011). This theory rests on two cornerstones. The first is that the difficulty of the task should be just right to promote optimal learning; not a routine task but also not too far beyond the learner’s skill level. The second is that learning is largely a social process. To solve and learn from the more difficult tasks, a supervisor is needed.

A general observation in our interviews was that the same occupation in the same position may include various conditions for decision latitude. Differences in work organization between day and night shifts influenced all aspects of the results: learning new things, opportunity to use skills and creativity, repetitiveness, and the freedom to decide what to do and how to do it. The RN’s role in the night organization was, compared with the day shift, described by the interviewees as far more autonomous and less fragmented, although at times with less access to support.

### Limitations

The interviews were not done with a particular focus on decision latitude, which can be both a limitation and an advantage. The limitation includes the risk that we may have neglected to appropriately deepen the interview. As an advantage, we have received a broad picture of what the interviewees’ work entails. Furthermore, we did not assess self-reported decision latitude, and consequently we cannot draw any conclusions about the level of decision latitude, or strain, in the study group. We strived for rigour in the processes for data collection and analysis, to optimize the interpretive validity of the findings. Notably, in qualitative research the researchers use their subjectivity in the process, which is seen as a strength rather than a weakness. Consequently, the details of the findings would likely be somewhat different with other interviewees and other researchers.

## Conclusions

Conclusions from the present study concern the contextual dependence of decision latitude. The present findings does not imply that decision latitude in other occupations would be affected by the same factors or in the same way as presented here. Rather, the primary message is that decision latitude appears to be influenced by the specific work organization. The nature of this influence in a particular occupation needs to be investigated. In other words, when we—in practice or research—address self-reported decision latitude, it can have different meanings and potentially different effects in different contexts.

Moreover, our findings indicate that the interface and interplay between the three constructs decision latitude, demand and support is relevant and very complex. For example, access to support may be critical to whether autonomy and challenging tasks involve learning or demands. Likewise, challenges that match one’s competence can provide job satisfaction, while challenges too far beyond one’s competence are perceived as demands. Workplace interventions intended to strengthen decision latitude should be encouraged to pay attention to this.

Finally, it is justified to assume that the relationship between decision latitude and well-being is complex. This applies to the individual case and may also be worth considering when assessing strain and iso-strain on a group level. Further research is needed to shed light on the details in this relationship, to contribute to making the DCS model even more relevant to modern organizations. Future studies could also pay attention to whether and, if so, how different work-related individual factors can affect the appreciation of decision latitude, e.g., experience in the profession.

## Data Availability

The data (interviews/transcripts) are not available via any public data repository.

## Geolocation information

The research was carried out in the Stockholm region, Sweden.
